# Developing a Measure for the Accuracy of Symptom Perception: The Congruence Between Self-reported Dyspnea and Physiological Parameters in the Dutch Lifelines Cohort Study

**DOI:** 10.1097/PSY.0000000000001382

**Published:** 2025-03-10

**Authors:** Aranka V. Ballering, Saya Niwa, Omer Van den Bergh, Judith G.M. Rosmalen

**Affiliations:** Department of Psychiatry University Medical Center Groningen, University of Groningen, Groningen, The Netherlands (Ballering, Rosmalen); Department of Clinical Neuroscience, Wrocław University of Science and Technology, Wrocław, Poland (Niwa); Faculty of Psychology and Educational Sciences, University of Leuven, Health Psychology, Leuven, Belgium (Van den Bergh); Internal Medicine, University Medical Center Groningen, University of Groningen, Groningen, The Netherlands (Rosmalen)

**Keywords:** predictive processing framework, symptom perception, epidemiology, public health, respiratory symptoms, sex differences, **ACP** = actual category probability; **CVD** = cardiovascular disease; **FEV1** = Forced Expiratory Volume in 1 second; **FEV1%pred** = Predicted Forced Expiratory Volume in 1 second; **FVC** = Forced Vital Capacity; **GAD** = generalized anxiety disorder; **IQR** = interquartile range; **LTE** = list of threatening experiences; **M** = mean; **Med** = median; **NA** = negative affect; **PA** = positive affect; **PANAS** = Positive and Negative Affect Scale, **PCA** = principal component analysis, **PD** = panic disorder; **SCL-90 SOM** = Symptom CheckList-90 Somatization

## Abstract

**Objective:**

We aimed to assess whether a symptom perception accuracy measure can be derived from routinely collected general population cohort data.

**Methods:**

We combined information on self-reported dyspnea and physiological parameters (FEV1%pred, body weight) from the Lifelines Cohort Study (*N*=138,594; 59.0% female; mean age=42.3 years [SD=11.0]) to obtain a symptom perception accuracy measure. Dyspnea was operationalized via the SCL-90 SOM subscale item. Using principal component analysis of available psychosocial variables known to correlate with symptom perception, we derived 3 compound scores reflecting negative affect, fear of illness, and worries of contracting disease. We used multinominal regression analyses to calculate the probability of self-reported dyspnea being correctly classified based on FEV1%pred and body weight. Via multivariable logistic regression we assessed whether the dichotomized probability of correct classification is associated with derived compound scores.

**Results:**

The symptom perception accuracy measure was non-normally distributed in control and participants with asthma/COPD. Fear of illness (OR=0.85; 95% CI=0.79-0.90 and OR=0.84; 95% CI=0.72-0.98) was negatively associated with the accuracy measure in control and asthma/COPD participants, respectively. Negative affect (OR=0.76; 95% CI=0.65-0.90) was associated negatively with the accuracy measure in asthma/COPD participants. Worries about contracting disease were associated with the measure in control participants (OR=0.88; 95% CI=0.83-0.94). Physiological parameters explain 1.6% to 2.5% of the variance in self-reported dyspnea; the addition of aforementioned compound scores increases this to 9.5% to 16.6%.

**Conclusions:**

We show that a symptom perception accuracy measure based on congruence between physiological parameters (FEV1%pred, body weight) and self-reported dyspnea can be developed. It is associated with known psychosocial correlates of symptom perception. The psychosocial factors explained more variance in self-reported dyspnea than physiological parameters.

## INTRODUCTION

People differ in their experience of symptoms in the presence of pathological processes.^1,2^ In other words, often a discrepancy exists between objective bodily assessments of dysfunction or pathology and the self-reported symptoms that people experience due to these. This discrepancy has been found across multiple medical domains, such as orthopedics, diabetology, and pulmonology. A systematic review including 33 studies showed that disk degeneration, which is frequently associated with back pain, occurred in 37% of pain-free individuals aged 20 to 30, increasing to 96% of pain-free individuals aged 80 or older.^3^ Conversely, improved glycemic control was not associated with a reduction of experienced diabetes-related symptoms.^4^ Pulmonary function, measured by Forced Expiratory Volume in one second (FEV1) as a percentage of predicted FEV1 was poorly correlated to the frequency and severity of asthma-related symptoms as well, despite its frequent use in clinical asthma studies.^5^ These examples show that pathology does not necessarily linearly correlate with symptom experience.

It is pivotal to obtain insights into the factors that determine people’s symptom experience, especially since one’s quality of life,^6^ but also treatment decisions depend on the severity of the symptoms reported by the patient. Previous studies suggested that the experience of somatic symptoms is the result of a multifaceted process, composed of interacting biomedical, psychological, and social factors. Factors contributing to symptom experience include, but are not limited to genotype,^7^ female sex,^8,9^ older age,^10^ the presence of (chronic) disease,^11^ the extent of neurotic traits,^12^ fear of illness,^13^ negative affect (NA),^14,15^ stress induced by long-term difficulties experienced in, for example, relationships, finances, or religious settings,^16^ life events,^16^ and feminine gender roles.^17^ These factors are all involved in a complex interplay of biological, psychological, and social processes.

Recent theories shed light on this complex interplay. The symptom perception perspective states that somatic symptoms are the outcome of perceptual-cognitive processes, including the generation and detection of a bodily signal, attention toward a bodily sensation, and interpretation of the signal into either a symptom or a passing sensation.^18^ The predictive processing theory has recently gained more attention and provides a conceptual framework for how pre-existing knowledge and experiences shape one’s perception of bodily sensations.^19^ It explains how the perception of symptoms is the result of a process in the brain that balances sensory information, and predictions regarding this sensory information, which are shaped by life experiences and beliefs about symptoms.

The influence of pre-existing knowledge and experiences on perceiving bodily sensations and symptoms is corroborated by experimental studies. For example, when 64 participants were asked to rate the intensity of thermal stimuli, it was found that induced negative expectations before the assessment increased participants’ perceived thermal intensity.^20^ Neuroticism and absorption (ie, the tendency to become involved in both imaginative and sensory experiences) were found to be associated with increased perceived thermal intensity as well. Similarly, another experimental study including 74 female participants undergoing the Standard Rebreathing Test showed that negatively framing experimental conditions induced higher respiratory symptom proneness, especially in participants who reported relatively high numbers of symptoms before the commencement of the study.^21^ Additional experimental studies have illustrated how contextual cues may affect one’s symptom proneness.^22,23^

Despite their high internal validity, these experimental studies are conducted in relatively small study samples. Currently, no measures for the accuracy of somatic symptom perception exist for use in large-scale cohort studies. In the current study, we define the accuracy of symptom perception as the degree of congruence between assessed pulmonary function and self-reported respiratory symptoms.

The lack of easy-to-use measures for the accuracy of somatic symptom perception hinders the possibility of conducting longitudinal studies on factors influencing symptom perception processes. The inclusion of a measure for the accuracy of somatic symptom perception in cohort studies would allow for studying elements of the predictive processing theory throughout the life course, the experiences that shape symptom perception, and their interaction with genetic factors contributing to symptom perception. However, the design and sample size of cohort studies are barriers to the inclusion of experimental approaches that are typically used to assess and develop measures of accuracy of symptom perception.

We aimed to explore whether a measure for individuals’ accuracy of symptom perception could be derived from routinely collected data in large-scale cohorts. To this end, we aimed to combine information on participants’ self-reported respiratory symptom experience, as well as pulmonary function. We focus on the respiratory system since high levels of respiratory symptom experience are relatively strongly associated with high levels of somatic symptom experience in general.^2^ Furthermore, respiratory symptoms are relatively directly correlated to one underlying organ system, that is readily and noninvasively assessed by spirometry. We explore the conceptual meaningfulness of a measure for the accuracy of respiratory symptom perception by assessing its associations with known correlates of symptom perception, including sex, neuroticism, NA, and fear of illness.

## METHODS

### Lifelines Cohort Study

This study is based on data collected within the Lifelines Cohort Study. The Lifelines Cohort Study is a multidisciplinary prospective population-based cohort study examining in a unique 3-generation design the health and health-related behaviors of 167,729 persons living in the North of the Netherlands. It employs a broad range of investigative procedures in assessing the biomedical, sociodemographic, behavioral, physical, and psychological factors that contribute to the health and disease of the general population. Lifelines especially focus on multimorbidity and complex genetics. Extensive information on Lifelines’ cohort, design considerations, and recruitment procedures are provided elsewhere.^24–26^ The Lifelines Cohort Study is performed according to the principles of the Declaration of Helsinki and in accordance with UMCG’s research code. The Lifelines Cohort Study is approved by the Medical Ethical Committee of the University Medical Center Groningen, The Netherlands (2007/152).

### Selection of the Study Sample

We included participants aged 18 years or older who entered the Lifelines cohort study at baseline. As we aimed to obtain a study sample in whom respiratory symptoms are relatively directly associated with respiratory dysfunction, we excluded participants with cardiovascular disease (CVD), generalized anxiety disorder (GAD), and panic disorder (PD) from our analyses. Although these disorders also frequently present with dyspnea, their relationship with the results of pulmonary function tests is less straightforward.

CVD was defined based on self-report of a physician-confirmed arrhythmia or aneurysm, angioplasty, or self-reported atherosclerosis, a heart attack, heart failure, heart valve problems, a stroke, or stenosis as assessed by questionnaires. If the participant indicated the presence of at least one of the aforementioned CVD, we considered them as having CVD. GAD and PD were defined by assessing the respective items from the Mini-International Neuropsychiatric Interview (MINI).^27,28^ In accordance with DSM-IV-TR duration criteria, GAD and PD were considered present if the participant indicated the presence of the required symptoms in the past 6 months and 1 month, respectively.^29^ We also excluded participants who self-reported an asthma diagnosis that was not confirmed by a physician to avoid potential misclassification. This resulted in the exclusion of 573 participants from the study.

### Pulmonary Function

Participants’ objective pulmonary functioning was assessed using spirometry performed by trained Lifelines assistants between November 2006 and December 2013. Spirometry was conducted according to the American Thoracic Society (ATS) guidelines, using the Welch Allyn SpiroPerfect device. Technically invalid spirometry measures are excluded from the study sample. Technically invalid spirometry was defined based on the evaluation of a trained professional of whether the spirometry procedure was conducted according to the ATS guidelines or is clinically reliable. Further details on spirometry are provided elsewhere.^30^ We used the FEV1% predicted (FEV1%pred) as indicator of pulmonary functioning. The FEV1%pred was calculated per individual (minimum-maximum: 16% to 225%). FEV1%pred represents the ratio of Forced Expiratory Volume in one second (FEV1) to Forced Vital Capacity (FVC) in liters, also referred to as FEV1%, divided by the average FEV1% based on participants’ age, sex, and height. The values of the Global Lung Functioning Initiative were used as reference values.^30^ FEV1%pred is a widely used spirometric parameter in both research and clinical settings.^31,32^ An increase in FEV1%pred indicates an increased pulmonary function.

### Chronic Pulmonary Diseases, Common Somatic Symptoms, and Dyspnea

We defined the presence of chronic pulmonary diseases, specifically asthma and COPD, based on self-reported diagnosis. We only included asthma diagnoses if these were confirmed by a physician, as reported by the participant. Information on self-reported physician-confirmed COPD diagnoses was not available.

Participants’ somatic symptom experience was assessed by the 12-item ordinal Symptom CheckList-90 Somatization (SCL-90 SOM) subscale, which has been validated and is recommended for use in large-scale studies.^33^ The SCL-90 SOM subscale assesses the extent of bother participants experience due to common somatic symptoms in the past week, including headaches, difficulties breathing, and general tiredness. It uses a 5-point Likert scale ranging from “1—not at all” to “5—extremely” (Appendix A, Supplemental Digital Content, http://links.lww.com/PSYMED/B82). The SCL-90 sumscore, ranging from 12 to 60, is constructed by adding the scores of the 12 included individual common somatic symptoms.

The SCL-90 SOM includes an item on dyspnea (ie, How much were you bothered in the past week by difficulties in breathing?), which was used as one of the self-reported dyspnea measures in this study. A second, self-reported measure of perceived dyspnea was calculated by summing the score of 5 general items that assessed experienced dyspnea dichotomously (0—no; 1—yes). These items were specifically designed for the purpose of the Lifelines study and were not validated. The items include whether (1) someone becomes short of breath when they lie (too) flat; (2) someone at times wakes up at night with shortness of breath; (3) someone ever had an attack of shortness of breath during daytime while at rest; (4) someone ever had an attack of shortness of breath after exertion and; (5) whether someone has ever woken up from an attack of shortness of breath.^34^ The dyspnea sumscore ranges from 0 to 5.

### Psychosocial Correlates of Symptom Perception

#### Life Events and Long-term Difficulties

The self-reported presence of negative life events in the past year was assessed based on the Dutch version of the List of Threatening Experiences (LTE), which comprises 12 items that can be answered dichotomously (eg, Could you indicate whether you experienced this unpleasant event in the last year?/You were severely ill, severely injured or a victim of violence). The LTE score comprises the sum of experienced negative life events (range 0 to 12).^16^

The Long-term Difficulties Inventory consists of 12 items referring to aspects of life, including housing, professional life, social relationships, leisure time, financial stability, and health (eg, In the past year, to what extent did you experience difficulties and stress related to this aspect of your life?/home and living, eg, accommodation too small, could not find a home, noise).^16^ Participants could indicate to what extent they experienced difficulty and stress on a 3-point scale ranging from 0—not stressful to 2—very stressful.

#### Neuroticism and Fear of Illness

We assessed the presence of neuroticism by calculating the sumscore of the NEO-PI-R neuroticism domain. The 6 facets of neuroticism include anxiety, hostility, depression, self-consciousness, impulsiveness, and vulnerability to stress. Each facet is surveyed by eight items, and can be answered on a 5-point Likert scale ranging from “1—strongly disagree” to “5—strongly agree.”^35^

Participants’ fear of illness was assessed by a validated Dutch version of the Whitely index.^13^ This index contains 14 true-false statements about one’s perception of health, for example, worries about the possibility of one having a serious illness or whether it is hard to believe for one when being told by a doctor there is nothing to worry about. We calculated a mean score, with a higher score indicating an increased fear of illness.

#### Positive and NA

Positive affect (PA) and NA were operationalized via the mean scores of the 10-item subscales of the validated Dutch version of the Positive and Negative Affect Schedules (PANAS).^36^ PA and NA are not 2 opposite poles of one dimension but are rather 2 distinct affective state dimensions that may co-occur. In other words, a lack of PA does not necessarily indicate the presence of NA, and vice versa. The items of the PANAS assess the extent one has felt positive and negative emotions over the past 4 weeks. This is measured on a 5-point Likert scale, ranging from “1—not at all” to “5—extremely.”

### Covariates

Participants’ ages in years and sex in a male/female binary were derived from the municipal database. As no information on gender identity is present, we refer to participants as male and female. Participants’ educational level was reported on a questionnaire, and subsequently categorized into a low, medium, and high level, as previously adhered to in Lifelines studies.^37^ Body weight was self-reported in kilograms. Smoking was reported on a questionnaire and dichotomized into a variable that assesses whether participants currently smoke or have smoked in the past month versus not currently smoking or did not smoke in the past month. Participants’ physical functioning and emotional well-being were operationalized by calculating participants’ self-reported mean score of the 10 items on the physical functioning and of the 5 items on the emotional well-being subscale of the RAND-36.^38^

### Statistical Analyses

Statistical analyses were conducted in IBM SPSS v.27. We calculated descriptive statistics of participants’ characteristics at baseline. For descriptive purposes, we dichotomized the dyspnea item derived from the SCL-90 SOM subscale into absent (score: 1) or present (score: ≥2).^39^ Normally distributed continuous variables were provided as mean, accompanied by a SD, whereas non-normally distributed variables were provided as median and interquartile range (IQR). To assess monotonous coherence between FEV1%pred, self-reported dyspnea operationalizations, and the SCL-90 SOM sumscore we calculated Spearman correlation.

To obtain a measure for the accuracy of symptom perception, we combined physiological measures, including FEV1%pred and body weight, with self-reported dyspnea measures. We accounted for body weight alongside FEV1%pred in our analyses, because body weight has been shown to affect respiratory symptoms and pulmonary functioning,^40^ but it is not accounted for in FEV1%pred. As a first step, we conducted multiple ordinal regression analyses, which assessed the association between multiple independent variables and an ordinal dependent variable. The independent variables included FEV1%pred and body weight. The dependent variable was experienced dyspnea, operationalized via the five-level ordinal SCL-90 dyspnea item. However, the assumption of proportional odds was not met (Χ_(df=3)_=10.5, *p*=0.015 for female participants; Χ_(df=3)_=10.4, *p*=0.015 for male participants). This indicates that the associations between the independent variables and the dependent variable are not equally strong across all levels of the dependent variable and, thus, that a single equation cannot capture the associations between the independent and dependent variables.

Therefore, we fitted a less restrictive multinomial logistic model. This model fits multiple equations over the levels of the dependent variable instead of a single equation. Again, the independent variables were FEV1%pred and body weight, while the dependent variable was dyspnea operationalized via the 5-level ordinal SCL-90 dyspnea item. This model was separately fitted for male and female participants and for participants with COPD/asthma and healthy controls as well.

We report the actual category probabilities (ACP) from the multinominal model that included FEV1%pred and body weight to predict dyspnea. This model was fitted for the control and asthma/COPD population separately. In these analyses, we only used the SCL-90 dyspnea item, as this is an item that is easily incorporated and reproduced in large-scale cohort studies, while dyspnea assessed by a sumscore consisting of multiple items is not easily available. The ACP represents the estimated probability that the underlying algorithm accurately classifies participants according to their reported dyspnea level based on the independent variables (ie, FEV1%pred and body weight). The ACP is central to the symptom perception accuracy measure we aimed to develop. As the frequency distribution of the ACP was not normally distributed in both the control and asthma/COPD populations, we dichotomized the ACP by performing a split on the respective medians. This allowed us to demarcate a category of participants whose pattern of physiological parameters could more accurately indicate their self-reported dyspnea, in contrast to a category of participants whose physiological parameters were less accurately indicating the participant’s experienced dyspnea.

Principal component analysis (PCA) was conducted to identify underlying components of the aforementioned psychosocial variables to reduce the number of variables and to be able to test broader dimensions. We included the sumscores of the Long-term Difficulties Inventory, LTE, the neuroticism-specific facets of the NEO-PI, the individual NA-related items of the PANAS, and individual items of the Whitely Index in the PCA. The suitability of PCA was assessed by calculating the Kaiser-Meyer-Olkin (KMO) coefficient. A KMO coefficient of >0.7 is considered adequate. The significance of the Bartlett test of sphericity indicates sufficient correlation between the items to perform PCA. We conducted PCA with oblique (oblimin) rotation as we expected correlations between the psychosocial variables. Standardized compound scores per obtained component were calculated. Items that did not load on a single component with a coefficient ≥0.4 or that loaded on more than one component with a coefficient ≥0.4 were removed before the calculation of standardized compound scores.^41^ To assess the symptom perception measure’s associations with known psychosocial correlates of symptom perception, we fitted a binary logistic model. Herein, we stepwisely included sex and the compound scores derived from the PCA as independent variables, while the median-splitted ACP was the dependent variable. We also assessed interaction terms between compound scores and sex to assess whether the association between psychosocial predictors and the congruence between pulmonary function and body weight, and self-reported dyspnea differs between male and female participants.

Last, we build a multinominal model with self-reported dyspnea, operationalized by the SCL-90 SOM item, as an outcome. Starting with FEV1%pred, body weight, and smoking, we stepwisely added the compound scores derived from the PCA as independent variables to the model. We repeated these analyses with the dyspnea sumscore and SCL-90 SOM sumscore as dependent variable, via multiple linear regression analyses. We report the variance in self-reported dyspnea operationalizations explained by the aforementioned independent variables to assess the extent to which physiological and psychosocial measures are able to explain the variation in self-reported dyspnea.

### Data Sharing

Lifelines data will not be shared publicly. Access to the Lifelines data is organized according to a strict data access procedure. For all types of access, a research proposal must be submitted for evaluation by the Lifelines Research Office. The evaluation is performed to align the goals of the researchers with the goals of Lifelines (which are in turn aligned with the informed consent form signed by Lifelines participants). Further information on Lifelines data can be obtained by contacting the Lifelines Research Office (https://www.lifelines.nl). Analysis code is available from the first author upon reasonable request.

## RESULTS

In total, we included 138,594 participants (59.0% female, mean age 42.3 [SD=11.0]). 21.5% (*N*=29,779) of the participants were smokers during the time of assessment, and 8.8% (*N*=12,171), 5.0% (*N*=6987), and 9.2% (*N*=12,796) of the participants indicated to have CVD, COPD, or asthma, respectively. In 103,252 (74.5%) participants, spirometry was performed, of which 97,511 (70.4%) participants had technically valid results. Dyspnea was experienced by 13,329 (9.6%) of the participants. Table 1 describes the characteristics of the participants stratified in a control population and population with asthma/COPD. Appendix B in the Supplemental Digital Content, http://links.lww.com/PSYMED/B82, describes the characteristics of participants stratified by sex. In total, 12.7% (*N*=17,601; 62.3% female) of participants reported CVD, PD, or GAD. These participants were excluded from further analyses.

**TABLE 1:** Overview of Participants’ Characteristics at Baseline

	Control participants (*N*=121,843; 87.9%)	Participants with asthma or COPD (*N*=16,751; 12.1%)
Female participants, N (%)	71,674 (58.8)	10,158 (60.6)
Age, M (SD)	42.6 (11.0)	41.7 (11.4)
Educational attainment, N (%)		
Low	33,117 (27.2)	5,179 (30.9)
Medium	49,853 (40.9)	6,885 (41.1)
High	37,719 (31.0)	4,499 (26.9)
BMI, M (SD)	25.8 (4.24)	26.7 (4.93)
Current smoker, *N* (%)		
Yes	25,992 (21.3)	3,787 (22.6)
No	91,424 (75.0)	12,180 (72.7)
Physical functioning, Med (IQR)	95.0 (90.0-100.0)	95.0 (80.0-100.0)
Emotional functioning, Med (IQR)	60.0 (56.0-64.0)	60.0 (56.0-64.0)
Positive affect, M (SD)	3.54 (0.42)	3.54 (0.45)
Negative affect, M (SD)	2.07 (0.53)	2.13 (0.56)
SCL-90 SOM score, M (SD)	1.34 (0.36)	1.48 (0.46)
Presence of dyspnea, N (%)^a^	8676 (7.1)	4,653 (27.8)
Presence of CVD, N (%)	10,251 (8.4)	1920 (11.5)
Presence of GAD, N (%)	5038 (4.1)	1096 (6.5)
Presence of PD, N (%)	280 (0.2)	63 (0.4)
Technically valid spirometry, N (%)	85,720 (70.4)	11,791 (70.4)
FEV1 in liters, M (SD)^b^	3.58 (0.82)	3.33 (0.85)
FVC in liters, M (SD)^b^	4.61 (1.03)	4.47 (1.04)
FEV1%pred, M (SD)^b^	96.7 (12.2)	85.3 (14.3)

^a^ Presence of dyspnea was defined as ≥2 on the SCL-90 dyspnea item.

^b^ Values shown are based on technically valid spirometry measures.

### Actual Category Probability (ACP) as a Measure of Accuracy of Respiratory Symptom Perception

**FIGURE 1:**
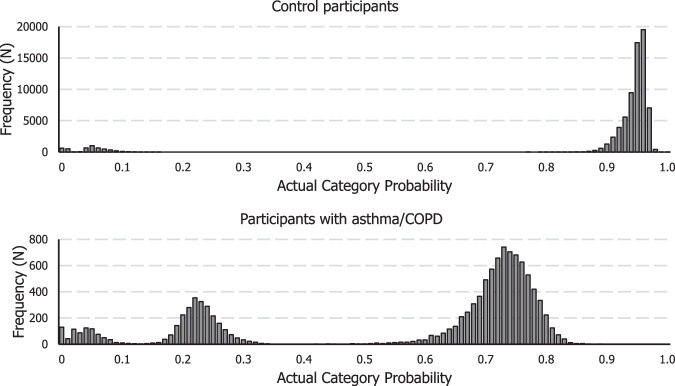
Frequency distribution of ACP in control participants and participants with asthma/COPD.

To obtain a measure of respiratory symptom perception, we performed a multinominal regression with physiological measures (ie, FEV1%pred and body weight) as predictors of self-reported dyspnea operationalized by the SCL-90 SOM item. This allowed us to calculate participants’ ACP, which constitutes the probability that participants’ self-reported dyspnea is accurately classified based on physiological variables. Participants’ ACP was non-normally distributed (Fig. 1). This indicates that in a proportion of the participants, patterns of FEV1%pred and body weight are a more accurate predictor of self-reported dyspnea than in others.

ACPs varied between the control participants and participants with asthma/COPD, with higher probabilities for correct classification of dyspnea based on pulmonary function and body weight in control participants than in participants with asthma/COPD. The median ACP in control participants was 0.94 (IQR=0.93 to 0.95). In total, 6.7% of the control participants had an ACP below 0.5. The low ACP indicates that the algorithm would not have classified these participants in their self-reported dyspnea category. The median ACP in participants with asthma/COPD was 0.70 (IQR=0.19 to 0.74), with 29.1% having an ACP of 0.5 or below. ACP and self-reported dyspnea only weakly correlate in control participants and participants with asthma/COPD (ρ=−0.27, *p*<.001; ρ=−0.37, *p*<.001, respectively). This implies that the accuracy of estimating participants’ dyspnea based on FEV1%pred and body weight, does not strongly correlate with self-reported dyspnea in control participants and participants with asthma/COPD.

### Association of the ACP With Psychosocial Components

We performed PCA on a series of available psychosocial components to define a comprehensive, albeit reduced, set of variables. The KMO coefficient was 0.915, and Bartlett test of sphericity provided a *p*<0.01, indicating sampling adequacy for the PCA. Based on the PCA, we retrieved components underlying the psychosocial variables of NA, fear of illness, long-term stressors, and experienced life events (Appendix C, Supplemental Digital Content, http://links.lww.com/PSYMED/B82). The 3 components include a broad NA component (Cronbach α=0.85), a broad fear of illness component (Cronbach α=0.75), and a component that reflects a worry of contracting disease (Cronbach α=0.60). The components explained 24.8%, 8.9%, and 7.4% of the total variance in the PCA input variables, respectively.

We next assessed whether these psychosocial components were associated with the measure of accuracy of symptom perception. In multiple logistic regression analyses, we found that male control participants have 0.61 (95% CI=0.54-0.69) times the odds of female control participants having a high ACP (Appendix D, Supplemental Digital Content, http://links.lww.com/PSYMED/B82). Fear of illness is negatively associated with the accuracy measure of symptom perception, in both healthy participants and participants with asthma or COPD (OR=0.85; 95% CI=0.79-0.90 and OR=0.84; 95% CI=0.72-0.98). In participants with asthma or COPD, NA was also negatively associated with the accuracy of symptom perception (OR=0.76; 95% CI=0.65-0.90). We also show the sex-stratified associations in Appendix D, http://links.lww.com/PSYMED/B82. These show, for example, that an increase in NA was associated with a low ACP in female participants with asthma/COPD (OR=0.72; 95% CI=0.58-0.89).

### Variance of Perceived Dyspnea and Somatic Symptoms Explained by Physiological and Psychosocial Measures

We aimed to explore whether the addition of psychosocial components to physiological parameters could improve the prediction of self-reported somatic, including respiratory, symptoms. Therefore, we first calculated the correlation between FEV1%pred, the dyspnea operationalizations, and SCL-90 SOM sumscore (Appendix E, Supplemental Digital Content, http://links.lww.com/PSYMED/B82). The correlations between FEV1%pred and dyspnea operationalizations show an inverse, weak correlation, indicating that an increase in FEV1%pred weakly correlates to a decrease in dyspnea.

Thereafter, we assessed the cumulative explained variance of common somatic symptoms, and specifically perceived dyspnea, by physiological measures. Only a small proportion of the variance in dyspnea, operationalized by the SCL-90 SOM item or sumscore is explained by physiological measures (1.6% to 2.5% and 1.2% to 2.1%, respectively). These percentages range from 1.0% to 2.1% for common somatic symptoms. Stepwise addition of the components NA, fear of illness, and worries of contracting disease increased the explained variance in self-reported dyspnea and common somatic symptoms significantly (Table 2). However, the variance explained by the included variables is higher for common somatic symptoms than for dyspnea.

**TABLE 2:** Cumulative Explained Variance in Perceived Dyspnea and Somatic Symptom Experience

	Female Participants		Male Participants	
	Control (N=40,292) (%)	COPD or Asthma (N=7070) (%)	Control (N=29,854) (%)	COPD or Asthma (N=4572) (%)
Model 1: SCL-90 SOM dyspnea		Pseudo *R* ^2^		
FEV1%pred + body weight + smoking	1.6	2.4	1.9	2.5
Component —“Negative affect”	5.7	13.4	5.8	9.1
Component 2—“Fear of illness”	8.0	15.3	9.4	11.7
Component 3—“Worries of contracting disease”	8.7	16.6	9.5	14.3
Model 2: Dyspnea Sumscore		*R* ^2^		
FEV1%pred + body weight + smoking	1.7	1.2	2.1	1.5
Component 1—“Negative affect”	2.9	1.8	4.0	3.7
Component 2—“Fear of illness”	3.9	2.7	6.3	5.1
Component 3—“Worries of contracting disease”	5.1	6.2	6.4	5.2
Model 3: SCL-90 SOM sumscore		*R* ^2^		
FEV1%pred + body weight + smoking	1.5	2.1	1.0	1.4
Component 1—“Negative affect”	10.0	13.3	9.4	7.2
Component 2—“Fear of illness”	18.0	19.9	20.5	18.0
Component 3—“Worries of contracting disease”	21.3	24.5	21.6	20.1

Stepwise addition of the factors increased the fit of the models significantly (*p*<.05 for every model, assessed by ANOVA).

## DISCUSSION

In this study, we combined information on participants’ physiological parameters (FEV1%pred and body weight) and perceived dyspnea to assess whether a measure for individuals’ accuracy of symptom perception could be derived from routinely collected data in large-scale cohorts. The accuracy measure reflects the degree to which physiological parameters such as FEV1%pred and body weight could correctly classify participant’s self-reported dyspnea in both control participants and in participants with asthma or COPD. The measure for accuracy was negatively associated with known psychosocial components of symptom perception. This suggests that individuals with higher levels of these psychosocial components are more likely to be misclassified by physiological parameters only. In addition, the results indicated that psychosocial components explained more variance in self-reported symptoms than physiological parameters in both women and men.

### Limitations and Strengths

The results of this study should be interpreted in light of its limitations and strengths. The first limitation of this study is that a small proportion of the study sample reported dyspnea. An imbalance in the outcome variable of the multinominal regression could result in an underestimation of the predictive accuracy of FEV1%pred and body weight for people perceiving dyspnea. Consequently, this may result in selective misclassification in which the ACP of participants who perceive dyspnea is more frequently underestimated than in participants who do not perceive dyspnea. In this study, this bias is likely limited, as the absolute frequency of people perceiving dyspnea is still rather high. Furthermore, ACP only weakly to moderately correlates with perceived dyspnea. Another limitation of this study includes the use of the SCL-90 SOM subscale. The SCL-90 SOM assesses participants’ experiences in the past 7 days. Bother cannot necessarily be equated to the experience of symptoms. Nevertheless, the SCL-90 SOM subscale is validated for its use in reporting somatic symptoms in large-scale general population cohorts.^33^ The use of merely one item from the SCL-90 SOM may be considered a limitation as well, but it also suits our aim of assessing whether a potential symptom perception measure could be constructed with variables typically included in cohorts. Furthermore, the dyspnea sumscore, consisting of 5 dyspnea variables, did not yield better results than the operationalization of dyspnea via the SCL-90 SOM item. Another limitation involved the time frame between the spirometry assessment and self-reported dyspnea. This may be problematic, as participants’ dyspnea and spirometry results may change over time. In our study, the median time between spirometry and the dyspnea assessment was 1 month (IQR=0 to 1), which implies that the dyspnea assessment was performed close to the dyspnea assessment for most participants. Last, disorders beyond CVD, PD, and GAD may also present itself with dyspnea. We limited exclusions to participants with CVD, PD, and GAD, as these are well established in Lifelines. As a result, the study sample may comprise people whose dyspnea could be attributed to pathological processes unrelated to pulmonary function. This may have reduced the internal validity of the measure of accuracy of symptom perception. A major strength of this study is the avoidance of selection bias by using a large-scale cohort study to identify participants with and without dyspnea. Furthermore, the use of multiple operationalizations of dyspnea allowed for the exploration of the most suitable symptom measure, as well as for triangulation of research results.

### Comparison With Literature

We found that FEV1%pred only weakly correlated with self-reported dyspnea. Furthermore, the combination of smoking, FEV1%pred, and body weight explained little variance in self-reported dyspnea (1.6% to 2.9%). This suggests that self-reported dyspnea in the general population can only be marginally attributed to decreased pulmonary functioning as assessed by FEV1%pred. This finding is supported by previous studies that show that pulmonary abnormalities do not directly correlate to respiratory outcomes. For example, in persistent asthma patients (*N*=1748; 67.6% female), a weak and statistically nonsignificant correlation was demonstrated between pulmonary function and the frequency and severity of asthma-related symptoms.^5^ Research in 49,438 patients diagnosed with COPD (46.1% female), reported a weak correlation between FEV1%pred and dyspnea (Pearson *r*=−0.28).^31^ Another study in 2164 (34.7% female) clinically stable COPD patients showed weak correlations between FEV1%pred and respiratory outcomes, such as dyspnea, exercise capacity, and exacerbations.^32^ In a proportion of these COPD patients with severe airflow obstruction (ie, GOLD stage III and IV), little to no respiratory symptoms, exacerbations, or impaired exercise tolerance were reported. This shows that the experience of respiratory symptoms is highly heterogeneous among COPD patients, which parallels our findings in the general population. This shows that adequately capturing heterogeneity in perceived respiratory symptoms requires more than spirometry.

The incongruence between (patho)physiology and reported symptoms is not only present in pulmonary diseases but also evident from the observation that musculoskeletal abnormalities are frequently found in asymptomatic individuals. For instance, meta-analytic evidence in 3110 asymptomatic participants shows that 30% of the 20-to-30-year-olds show a disc bulge when undergoing magnetic resonance imaging (MRI), increasing to 84% in people aged 80 or older.^3^ A meta-analysis including 3761 knees in 2817 asymptomatic participants reports a pooled prevalence estimate of meniscal tears of 4% in people aged under 40 and 19% in people aged 40 years and older.^1^ In light of these studies, the sole use of (patho)physiological measures, whether these comprise spirometry or MR images, does not suffice to explain symptom perception in general population cohorts.

In addition, we found that compound scores relating to NA, fear of illness, and worries about disease were significantly associated with the ACP. One may consider the ACP as a rough approximation of interoceptive accuracy. In line with our study, previous research has shown that an increase in negative affectivity is associated with reduced interoceptive accuracy: negative affective states weaken the association between objective indicators of bodily dysfunction (eg, spirometry) and experienced symptoms (eg, self-reported dyspnea).^42–44^ Our findings support a recent predictive processing perspective that states that symptoms are the result of a balancing process in which sensory information from the peripheral body is integrated with prior predictions from the brain. The eventual symptom report depends on the relative reliability of both sources of information: in conditions with strong (implicit) predictions and relatively weak sensory information, the experienced symptom will be closer to the predictions and will only be weakly associated with the sensory information. NA, fear of illness, and health-related worrying are sources of strong predictions while being associated with less detailed processing of the somatic input. As a result, experienced symptoms reflect predictions more strongly than sensory information from the peripheral body.^44,45^ This may give rise to a potential discrepancy between experienced symptoms and (patho)physiological bodily changes.^44^ The predictive processing perspective allows the understanding of how psychosocial factors may shape neurobiological processes associated with symptom perception.^46^ Our findings clearly demonstrate that the widespread notion of somatic symptoms being the sole result of physiological changes or bodily abnormalities is invalid.

### Recommendations for Future Research

This study shows that the usability of a measure for the accuracy of symptom perception based on the congruence between (patho)physiological parameters and self-reported respiratory symptoms remains limited. We therefore require the development of methods to assess individualized symptom perception in large cohort studies. Experimental cold pressor tests to assess pain tolerance have been included in large cohort studies, such as the Tromsø study,^47^ but these measures do not sufficiently capture participants’ symptom perception, especially in relation to respiratory symptoms or systemic symptoms, such as fatigue. If possible, future research should include large, heterogeneous study samples in which individuals are matched on (respiratory) symptom experience, age, ethnicity, sex, and comorbidities, allowing for more balanced groups in terms of demographic characteristics and health outcomes. Studies in longitudinal cohorts could reveal how biopsychosocial factors shape the accuracy of symptom perception throughout the life course. Better insights into these processes, preferably on an individualized level, allow for the development of personalized treatment strategies and may contribute to the prevention of symptom proneness.

## Supplementary Material
