# Supplementary material for: Developing a Measure for the Accuracy of Symptom Perception: The Congruence Between Self-reported Dyspnea and Physiological Parameters in the Dutch Lifelines Cohort Study
Source: Biopsychosoc Sci Med. 2025 Mar 10;87(4):249–58. doi: 10.1097/PSY.0000000000001382 (PMC12045335; doi:10.1097/PSY.0000000000001382)
Supplement: Supplementary file 1 [file psy-87-249-s001.docx]

***Appendix A***

The Symptom CheckList-90 somatization subscale (SCL-90 SOM) is part of the Symptom CheckList-90 (SCL-90). It assesses the extent of bothering participants experience due to common somatic symptoms. The total score may range from 12–60, whereas the mean score of the SCL-90 SOM may range from 1-5.

| **How much in the past week were you bothered by:** | | **Not at all** | **A little bit** | **Moderately** | **Quite a bit** | **Extremely** |
| --- | --- | --- | --- | --- | --- | --- |
| **1** | Headache | 1 | 2 | 3 | 4 | 5 |
| **2** | Dizziness | 1 | 2 | 3 | 4 | 5 |
| **3** | Chest pain | 1 | 2 | 3 | 4 | 5 |
| **4** | Lower back pain | 1 | 2 | 3 | 4 | 5 |
| **5** | Nausea | 1 | 2 | 3 | 4 | 5 |
| **6** | Painful muscles | 1 | 2 | 3 | 4 | 5 |
| **7** | Difficulties breathing | 1 | 2 | 3 | 4 | 5 |
| **8** | Feeling hot and cold alternately | 1 | 2 | 3 | 4 | 5 |
| **9** | Numbness or tingling in parts of your body | 1 | 2 | 3 | 4 | 5 |
| **10** | Feeling a lump in your throat | 1 | 2 | 3 | 4 | 5 |
| **11** | Weakness in body parts | 1 | 2 | 3 | 4 | 5 |
| **12** | Heavy arms or legs | 1 | 2 | 3 | 4 | 5 |

***Appendix B***

| ***Appendix B:*** Overview of participants’ characteristics at baseline, stratified by sex. | | | |
| --- | --- | --- | --- |
|  | | **Female participants (N=81,832; 59.0%)** | **Male participants (N=56,762; 41.0%)** |
| Period of data collection in calendar years | | 2006-2014 | 2006-2014 |
| Age, M (SD) | | 42.0 (11.1) | 42.7 (10.9) |
| Educational attainment, N (%) | Low | 22,164 (27.1) | 16,132 (28.4) |
|  | Medium | 34,499 (42.2) | 22,239 (39.2) |
|  | High | 24,370 (29.8) | 17,848 (31.4) |
| BMI, M (SD) | | 25.7 (4.8) | 26.3 (3.7) |
| Current smoker, N (%) | Yes | 16,336 (20.0) | 13,443 (23.7) |
|  | No | 62,337 (76.2) | 41,267 (72.7) |
| Physical functioning, Med (IQR) | | 95.0 (85.0-100.0) | 95.0 (90.0-100.0) |
| Emotional functioning, Med (IQR) | | 60.0 (56.0-64.0) | 60.0 (60.0-64.0) |
| Positive affect, M (SD) | | 3.54 (0.43) | 3.54 (0.43) |
| Negative affect, M (SD) | | 2.15 (0.53) | 1.97 (0.51) |
| SCL-90 SOM score, M (SD) | | 1.41 (0.4) | 1.28 (0.3) |
| Presence of dyspnea, N (%)^1^ | | 8,336 (10.2) | 4,993 (8.8) |
| Presence of pulmonary disease, N (%) | Asthma | 7,795 (9.5) | 5,001 (8.8) |
|  | COPD | 4,190 (5.1) | 2,797 (4.9) |
| Presence of CVD, N (%) | | 7,103 (8.7) | 5,068 (8.9) |
| Presence of GAD, N (%) | | 4,305 (5.3) | 1,829 (3.2) |
| Presence of PD, N (%) | | 240 (0.3) | 93 (0.2) |
| Technically valid spirometry, N (%) | | 57,757 (70.6) | 39,754 (70.0) |
| FEV1 in liters, M (SD)^2^ | | 3.1 (0.5) | 4.2 (0.7) |
| FVC in liters, M (SD)^2^ | | 4.0 (0.6) | 5.5 (0.83) |
| FEV1%pred, M (SD)^2^ | | 96.0 (12.4) | 95.9 (12.9) |
| ^1:^ Presence of dyspnea was defined as ≥2 on the SCL-90 SOM dyspnea item.  ^2:^ Values shown are based on technically valid spirometry measures | | | |

***Appendix C***

| **Appendix C.1:** Component loadings | | | |
| --- | --- | --- | --- |
|  | **Rotated component loadings (oblimin rotation)** | | |
|  | Factor | | |
|  | 1  “Negative affect” | 2  “Fear of illness” | 3 “Worries of contracting disease” |
| **Neuroticism domain** |  |  |  |
| Sumscore Anxiety Facet (NEO) | 0.793 |  |  |
| Sumscore Depression Facet (NEO) | 0.787 |  |  |
| Sumscore Hostility Facet (NEO) | 0.616 |  |  |
| Sumscore Impulsivity Facet (NEO) | 0.403 |  |  |
| Sumscore Self-consciousness Facet (NEO) | 0.657 |  |  |
| Sumscore Vulnerability Facet (NEO) | 0.709 |  |  |
| **PANAS – Negative affect items** |  |  |  |
| Frequency of feeling distressed | 0.625 |  |  |
| Frequency of feeling upset | 0.645 |  |  |
| Frequency of feeling guilty | 0.613 |  |  |
| Frequency of feeling scared | 0.732 |  |  |
| Frequency of feeling hostile | 0.494 |  |  |
| Frequency of feeling irritable | 0.570 |  |  |
| Frequency of feeling ashamed | 0.605 |  |  |
| Frequency of feeling nervous | 0.727 |  |  |
| Frequency of feeling jittery | 0.726 |  |  |
| Frequency of feeling afraid | 0.732 |  |  |
| **Whitely Index** |  |  |  |
| Worries about possibly having serious illness |  | 0.664^2^ | -0.380 |
| Suffering from aches and pains |  | 0.561 |  |
| Aware of things that happen in body |  | 0.409 |  |
| Worried about health |  | 0.727 |  |
| Having symptoms of very serious illnesses |  | 0.527 |  |
| Worrying about contracting disease after media attention |  |  | -0.566 |
| Irritation if someone tells you you look better if you feel ill^1^ |  |  |  |
| Suffering from many symptoms |  | 0.635 |  |
| Easily forgetting about oneself^1^ |  |  |  |
| Difficulties believing physicians |  | 0.413 |  |
| Feeling not being taken seriously |  | 0.459 |  |
| More worried about health than most people |  | 0.565 |  |
| Thinking something is seriously wrong with body |  | 0.610 |  |
| Afraid of disease |  | 0.394 | -0.524^2^ |
| **LDI** |  |  |  |
| Total amount of long-term stressors | 0.432 |  |  |
| **LTE** |  |  |  |
| Total number of negative life events^1^ |  |  |  |
| ^1^: Item did not load sufficiently on one of the three factors (values <0.4)  ^2^: Included in the factor in the respective column | | | |

**Appendix C.2:** Scree plot of principal component analyses


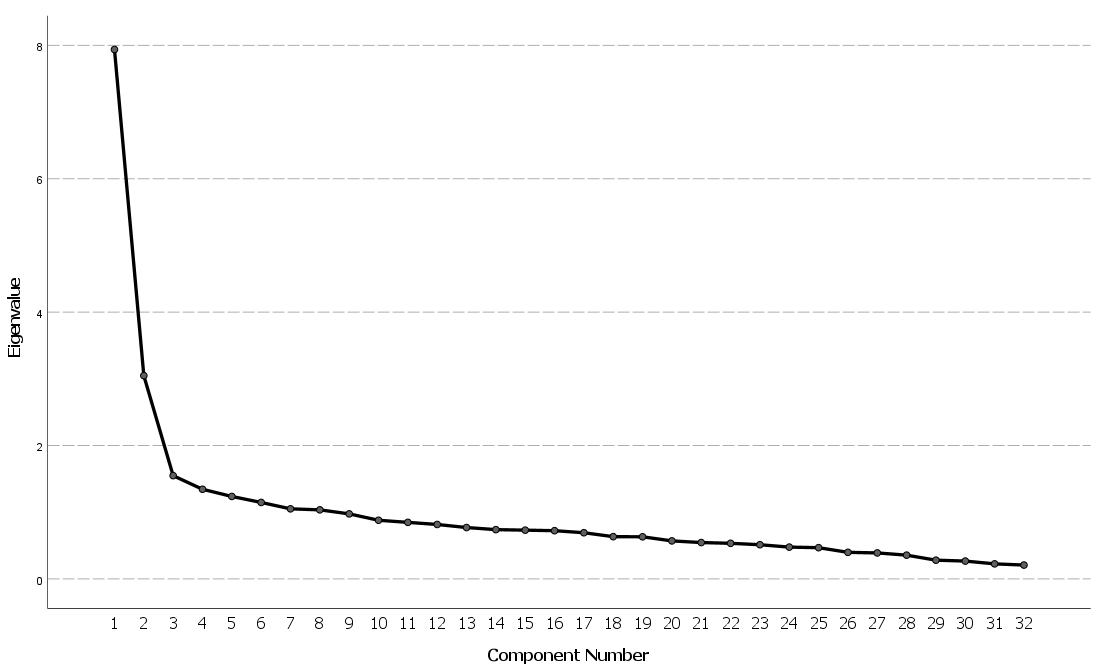


***Appendix D***

| **Appendix D.** Multivariable associations between derived psychosocial components and the ACP. | | | | | | |
| --- | --- | --- | --- | --- | --- | --- |
|  | **Full study sample** | | | | | |
|  | **Control participants** | | | **Participants with asthma/COPD** | | |
|  | β | *p* | OR (95%CI) | β | *p* | OR (95%CI) |
| Male sex | -0.49 | <0.001 | 0.61 (0.54-0.69) | -0.15 | 0.320 | 0.86 (0.65-1.15) |
| Component 1 – “Negative affect” | -0.06 | 0.081 | 0.95 (0.89-1.01) | -0.27 | 0.002 | 0.76 (0.65-0.90) |
| Component 2 – “Fear of illness” | -0.17 | <0.001 | 0.85 (0.79-0.90) | -0.18 | 0.022 | 0.84 (0.72-0.98) |
| Component 3 – “Worries of contracting disease” | -0.13 | <0.001 | 0.88 (0.83-0.94) | -0.12 | 0.140 | 0.90 (0.77-1.04) |
|  | **Female participants** | | | | | |
|  | **Control participants** | | | **Participants with asthma/COPD** | | |
|  | β | *p* | OR (95%CI) | β | *p* | OR (95%CI) |
| Component 1 – “Negative affect” | -0.06 | 0.142 | 0.94 (0.87-1.02) | -0.33 | 0.002 | 0.72 (0.58-0.89) |
| Component 2 – “Fear of illness” | -0.19 | <0.001 | 0.83 (0.76-0.90) | -0.21 | 0.043 | 0.81 (0.73-0.99) |
| Component 3 – “Worries of contracting disease” | -0.14 | <0.001 | 0.87 (0.81-0.94) | -0.07 | 0.472 | 0.93 (0.77-1.13) |
|  | **Male participants** | | | | | |
|  | **Control participants** | | | **Participants with asthma/COPD** | | |
|  | β | *p* | OR (95%CI) | β | *p* | OR (95%CI) |
| Component 1 – “Negative affect” | -0.05 | 0.353 | 0.95 (0.86-1.06) | -0.19 | 0.19 | 0.83 (0.63-1.10) |
| Component 2 – “Fear of illness” | -0.13 | 0.024 | 0.88 (0.79-0.98) | -0.27 | 0.018 | 0.76 (0.60-0.97) |
| Component 3 – “Worries of contracting disease” | -0.11 | 0.043 | 0.90 (0.81-1.00) | -0.17 | 0.159 | 0.84 (0.67-1.07) |

***Appendix E***

| **Appendix E**: Spearman’s correlations between FEV1%pred, dyspnea and SCL-90 SOM sumscores | | | | | | | | | | | | | | | | | | |
| --- | --- | --- | --- | --- | --- | --- | --- | --- | --- | --- | --- | --- | --- | --- | --- | --- | --- | --- |
| **Full study sample (N=97,511)** | | | | | | | | | | | | | | | | | | |
|  | **Control participants** | | | | | | **Participants with COPD** | | | | | | **Participants with asthma** | | | | | |
|  | SCL-90 dyspnea | | Dyspnea sumscore | | SCL-90 SOM | | SCL-90 dyspnea | | Dyspnea sumscore | | SCL-90 SOM | | SCL-90 dyspnea | | Dyspnea sumscore | | SCL-90 SOM | |
| FEV1%pred | ρ=-0.05 (p<0.001) | | ρ=-0.08 (p<0.001) | | ρ=-0.05 (p<0.001) | | ρ=-0.16 (p<0.001) | | ρ=-0.11 (p<0.001) | | ρ=-0.03 (p=0.037) | | ρ=-0.12 (p<0.001) | | ρ=-0.12 (p<0.001) | | ρ=-0.04 (p<0.001) | |
| SCL-90 dyspnea |  | |  | | ρ=0.28 (p<0.001) | |  | |  | | ρ=0.42 (p<0.001) | |  | |  | | ρ=0.47 (p<0.001) | |
| Dyspnea sumscore |  | |  | | ρ=0.28 (p<0.001) | |  | |  | | ρ=0.29 (p<0.001) | |  | |  | | ρ=0.28 (p<0.001) | |
| **Female participants (N=57,757)** | | | | | | | | | | | | | | | | | | |
|  | | **Control participants** | | | | | | **Participants with COPD** | | | | | | **Participants with asthma** | | | | |
|  | | SCL-90 dyspnea | | Dyspnea sumscore | | SCL-90 SOM | | SCL-90 dyspnea | | Dyspnea sumscore | | SCL-90 SOM | | SCL-90 dyspnea | | Dyspnea sumscore | | SCL-90 SOM |
| FEV1%pred | | ρ=-0.04 (*p*<0.001) | | ρ=-0.08 (*p*<0.001) | | ρ=-0.05 (*p*<0.001) | | ρ=-0.11 (*p*<0.001) | | ρ=-0.11 (*p*<0.001) | | ρ=-0.03 (*p*=0.07) | | ρ=-0.10 (*p*<0.001) | | ρ=-0.12 (*p*<0.001) | | ρ=-0.04 (*p*<0.001) |
| SCL-90 dyspnea | |  | |  | | ρ=0.27 (*p*<0.001) | |  | |  | | ρ=0.40 (*p*<0.001) | |  | |  | | ρ=0.42 (*p*<0.001) |
| Dyspnea sumscore | |  | |  | | ρ=0.28 (*p*<0.001) | |  | |  | | ρ=0.29 (*p*<0.001) | |  | |  | | ρ=0.24 (*p*<0.001) |
| **Male participants (N=39,754)** | | | | | | | | | | | | | | | | | | |
|  | | **Control participants** | | | | | | **Participants with COPD** | | | | | | **Participants with asthma** | | | | |
|  | | SCL-90 dyspnea | | Dyspnea sumscore | | SCL-90 SOM | | SCL-90 dyspnea | | Dyspnea sumscore | | SCL-90 SOM | | SCL-90 dyspnea | | Dyspnea sumscore | | SCL-90 SOM |
| FEV1%pred | | ρ=-0.05 (*p*<0.001) | | ρ=-0.10 (*p*<0.001) | | ρ=-0.04 (*p*<0.001) | | ρ=-0.14 (*p*<0.001) | | ρ=-0.13 (*p*<0.001) | | ρ=-0.04 (*p*=0.115) | | ρ=-0.15 (*p*<0.001) | | ρ=-0.15 (*p*<0.001) | | ρ=-0.05 (*p*=0.005) |
| SCL-90 dyspnea | |  | |  | | ρ=0.29 (*p*<0.001) | |  | |  | | ρ=0.48 (*p*<0.001) | |  | |  | | ρ=0.44 (*p*<0.001) |
| Dyspnea sumscore | |  | |  | | ρ=0.28 (*p*<0.001) | |  | |  | | ρ=0.27 (*p*<0.001) | |  | |  | | ρ=0.26 (*p*<0.001) |
| Note: the SCL-90 dyspnea item was not removed from the total score of the SCL-90 SOM subscale. | | | | | | | | | | | | | | | | | | |
